# Flow Analysis at the Snow Covered High Altitude Catchment via Distributed Energy Balance Modeling

**DOI:** 10.1038/s41598-019-39446-1

**Published:** 2019-03-18

**Authors:** Abdul Shakoor, Naeem Ejaz

**Affiliations:** Department of Civil and Environmental Engineering, University of Engineering and Technology, Taxila, 47070 Pakistan

## Abstract

Energy budget-based distributed modeling at high-altitude glacio-nival watersheds is essential to accurately describe hydrological processes and quantify the flow rates. In this study, SNOWPACK model and its distributed version Alpine3D are applied for the first time in Pakistan to simulate the runoff response of a high altitude glaciated catchment. The basic aim was to explore the feasibility of this modeling system and its future applications in the region. Final results demonstrated satisfactory performance of the model between measured and modeled discharges with Nash-Sutcliff Efficiency of 0.54. However, total simulated flow volume differs only 1.3 times as compared to measured discharge of the lake, located at the glacier snout. Flow composition analysis revealed that the runoff regime of the study site is strongly influenced by the snow and glacier melt runoff representing 53% snowmelt and 38% glacier melt contribution. Low model efficiency has been observed during glacier melting season due to inaccurate wind speed distribution and biased input met-data. It is concluded that high performance of this model can be achieved if the model is optimized over the catchment similar to the study site provided with long term data sets. This study leaves a firm foundation for the potential application of a highly accurate distributed energy balance model in the entire Karakoram and Himalaya region to understand the melt dynamics of such a rugged terrain glacier rich mountains.

## Introduction

The Hindu Kush, Himalaya and Karakoram are enriched with natural reserves of snow and glaciers which maintain water balance in this region and adjacent plains^1,2^. Snow and glacier melting in the Karakoram Range constitutes the major proportion of Indus river flow^3^. Pakistan is highly dependent on the flow from this river mainly for hydropower production and agricultural irrigation^4,5^. Quantitatively, Indus River System meets ninety percent of Pakistan irrigation needs and thirty percent of its total energy generation^6^. The agriculture-based economy of Pakistan relies on this river, by irrigating its arid lowlands^7^, and is considered as the back bone of Pakistan agricultural economy^8^. Contribution of agricultural economy to Gross Domestic Product is 21% in Pakistan that increases 2.7% every year^9^. Pakistan agricultural industry not only provides employment opportunities to 44% of the labors, it also fosters 62% of the rural population to enhance their livelihood^9^. Consequently, variability in the flow of this river impels profound effects upon the lives of people in Pakistan by direct or indirect ways^8^. Due to global warming, frozen water resources in this region are experiencing a decline in their mass swiftly raising the extent of glacial lakes and leading to the violent floods^10^. The increased temperature trends due to the climate change already enhanced melt rates and given rise to the dangerous lake formation process and outburst floods in northern parts of Pakistan^11^. The devastating hazards related to unexpected flow variations in these mountains have become more frequent during the recent years and surpassed all the past records^11^ claiming huge loss of lives and property damages. Pakistan is a home of approximately 5000 glaciers and 2500 glacial lakes located in 10 sub-basins^11^. However, considerable amount of uncertainties have been found in past hydrological studies in this region. These uncertainties are mostly related with the use of diverse modeling tools and limited quality input data as well as poor feedback mechanism^12^. Some studies suggests the increasing trend of glacier retreat in the Greater Himalaya and most of the area of mainland Asia in the recent past^13–15^ while other shows that many central Karakoram glaciers began advancing^16–19^.

It is therefore extremely essential to compute the accurate quantities of snow and glacier melt runoff to overcome the challenges of water resources management in these catchments. It is equally important to use the reliable method and tools for simulating melt and runoff dynamics in order to cope with the climate change impacts. Moreover, accumulation of snow and melt processes have to be expressed in a distributed way since the meteorological conditions and snow covered glaciers are subjected to intense temporal and spatial variations in the high-altitude catchments. In the past, hydrological studies related to the high altitude watersheds, both energy-balance and temperature-index models have been used to simulate melt rates^20–24^. But, due to the reduced computational time, input data requirement and easy to calibrate researchers prefer the use of conceptual models. Physics-based models are therefore avoided which requires immense effort to correctly extrapolate the meteorological parameters especially in high altitude complex mountainous terrains where meteorological and surface-atmospheric processes are highly variable. However, considering climate change, the use of temperature-index models is disputable since the value of the calibrated parameters required by these models may not be the same for all future climatic conditions^20^.

Likewise, hydrological models applied over upper Indus basin were mostly conceptual rather than physically and spatially distributed^25^. Research studies focusing on snow and ice melt modeling of high altitude catchments in Pakistan are even scarce due to lack of meteorological data, accessibility and resources. All except few studies are based on simpler conceptual models and uses lumped approach rather than spatially distributed to assess the hydrology and melt dynamics in present and future climate scenarios^2,3,12,26–29^. Moreover, hydrological models used in these catchments depict considerable variations in the outputs and therefore poses uncertainties. The uncertainties are mainly due to model parameters, climate modeling tools and inter-annual variations in temperature and precipitation due to climate change^12,30^. Lack of hydro-meteorological and cryospheric data mainly prevents to apply the more standardize techniques used in extensive data-driven models^12^. Data scarcity prevents systematic calibration procedures to identify optimized parameters set that could ensure internal consistency in the modeling of different hydrological processes^12^ Hydrological modeling is very challenging in such basins because of internal and external inconsistencies. For instance, underestimation in precipitation rates or biased input data can be counterbalanced by an overestimation of melting or energy balance of the study catchment considerably influenced by other high altitude complex phenomenon such as katabatic winds. These inconsistencies can covertly contribute towards bias melt and runoff generation and hidden simultaneously due to the complexity of feedback mechanisms in this region.

Present research uses point scale simulations to assess melt with a highly accurate energy balance model in order to compute the parameters of distributed hydrological model. This technique works by the concept that simulated melt rates with a physics based energy balance model are very precise at the location of Automatic Weather Stations due to the use of available high-quality meteorological measurements. Consequently, such simulation outputs can be used as a replacement of high-resolution ablation data and can be used to optimize parameters in the distributed hydrological model. It therefore enables to reproduce each melt process at different scales through given locations and lessen the uncertainties related to the model parameterization. Spatially and temporally robust model parameter values can be identified with only few seasons of available data^31^. These parameters are usually omitted in order to avoid the complexity of more standardize calibration procedures at a distributed scale. In this study, a highly sophisticated energy balance model (SNOWPACK) and its distributed version (Alpine3D) are applied for the first time in Pakitan at Passu catchment located in Hunza river basin. The aim was to check the feasibility of the model by quantifying the accurate flow composition and quantities and explore its capabilities for potential applications in future studies. The Himalaya and Karakorum regions are known for extremely intricate conditions and variable snow-atmospheric process^2^. Snow accumulation, melt and runoff generation and the overall response of glacio-nival watersheds is highly unpredictable here^2^. This model has shown good performance in the past from avalanche warning and hydrological studies to climate change^32–35^. Present research will evaluate its performance in the snow/ice covered rugged and steep terrain Pakistani catchments and explore the feasibility for entire Hindu Kush-Himalayan region as well as climate change studies. The model considers physical processes in a distributed hydrological modeling framework at a catchment scale to assess melting and would therefore diminish various sources of uncertainties. The study will also smooth grounds for the application of such modeling tool in future energy and mass balance studies in this region and will foster accurate forecast hydrological processes due to the impact of climate change.

## Site Description

**Figure 1:**
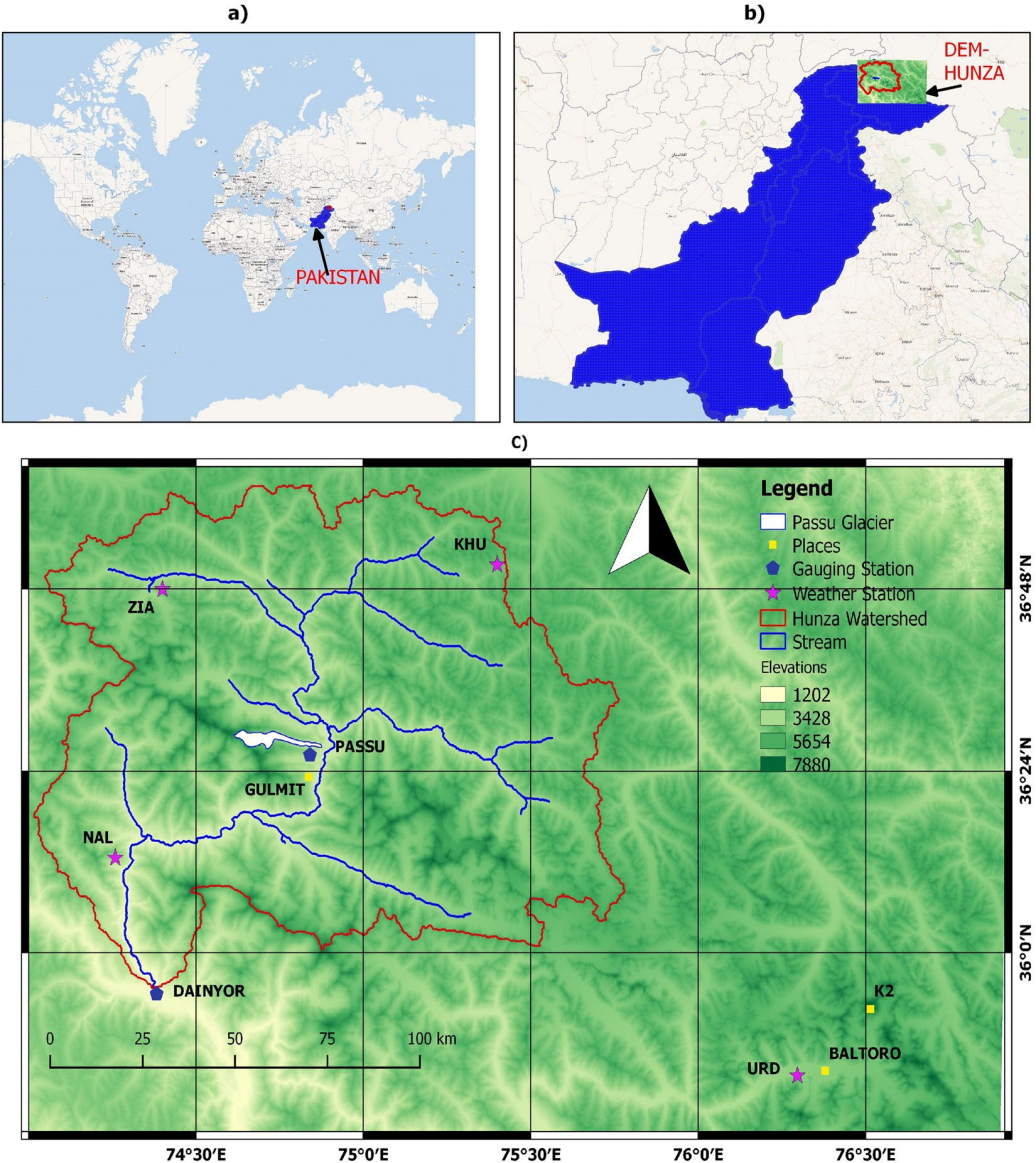
(**a**) Location of Pakistan in the world map (**b**) Location of Hunza basin in the Pakistan (**c**) Digital elevation model showing location and elevation of Hunza basin and other necessary details. (This map was made using QGIS software (version 2.18).

Passu glacier was chosen as a study site to perform this modeling test by analyzing melt and runoff, as it is located in climatically temperate region of the Himalayas and meet desired data availability. It is geographically situated at 36°27′ to 36°28′N and 74°38′ to 74°52′E in the south of Batura glacier. The elevation of Passu glacier rises from 1,200 m at the base to 7,500 m high peak above the mean sea level and contains steep slopes. It lies in Hunza river basin some 15 km from Gulmit, in the Gilgit-Baltistan region of Pakistan. It is about 150 km upriver from Gilgit and 15 km upstream of Ata-abad Lake^36^. This is a valley type glacier having length of 26 km and covers area of 63 sq. km with ice reserves nearly 10.89 cu. km. It feeds river Hunza, a tributary of the Indus river, in northern Pakistan which flows west to east^11,36,37^. Figure 1 shows the map of Hunza river basin and location of Passu Glacier along with nearby meteorological weather stations.

Passu glacial lake is located at the terminus of east-west oriented Passu glacier and receives water generated from the seasonal snow cover and glacier melt. A small stream arises from the lake which later joins the river Hunza which is a tributary of the Indus river^36^.

The villages located along the Khunzhrav river are at high risks of river floods especially in Gojal valley, Shimshal, Passu, Hussaini, Ghulkin, Gulmit^38^. Passu lake imparted two major outbursts floods in the last forty years with consequent downstream infrastructural damages which wiped out several houses of Passu village. The massive flow generated in these deadly events was associated with the occurrence of heavy rainfall in just 2 hours and accelerated melt rates due to 14 days long intense heat wave. These outburst events were mainly comprised of mud accompanying with a mixture of debris and boulders that rushed downstream and destroyed many structures on its path^11^.

## Model Description

Alpine3D is a spatially distributed energy balance model developed to predict the dynamics of snow-rich surface processes in the mountainous regions^39^. Alpine3D is based on the snow cover model named as SNOWPACK which resolves the partial differential equations by considering mass and energy balance and momentum conservation within the snowpack. This numerical model deals with one-dimensional processes and assumes that lateral fluxes are negligible compared to vertical exchanges^32^. Two boundary conditions control the net energy exchange at the snow-atmosphere interface namely (i) Neumann boundary condition (ii) Dirichlet boundary condition.

1 $${K}_{s}\frac{\partial {T}_{s}}{\partial z}{|}_{z=h,t}={Q}_{sh}+{Q}_{lw}+{Q}_{lh}+{Q}_{rr}$$

Equation 1 shows the first condition where, “K_s_” and “T_s_” are thermal conductivity and temperature of the snow respectively, at a given time “t”. “z” represents the perpendicular coordinate to the snowpack slope. When z = 0, model considers the snowpack base and as z = h it takes the snowpack surface for computations. Whereas, (Q_sh_) represents sensible heat exchange, (Q_lw_) represents the long-wave radiation, (Q_lh_) represents latent heat exchange and (Q_rr_) represents heat flux due to rain. When net heat exchange is positive, energy enters into the snowpack and if negative, energy is withdrawn^32^. Second possibility is the Dirichlet boundary condition shown in (Eq. 2)

2 $${K}_{s}\frac{\partial {T}_{s}}{\partial z}{|}_{z=h,t}={T}_{h}(t)$$

Where, T_h_ represents the temperature directly measured by automatic weather station. When melting starts, the temperature at the snowpack surface becomes 0 °C. Thus, dirichlet boundary condition cannot be used during ablation periods because it will underestimate the real energy input. Model therefore automatically changes it mode to run neumann’s boundary condition as soon as the temperature at the snowpack surface reaches the melting temperature^32^.

SNOWPACK model describes mass and energy exchange between the snow and atmosphere and also include the effects of vegetation cover and the soil optionally. This model was initially developed to support avalanche warning^33^, by providing a very detailed description of snow properties including weak layer characterization. It is successfully applied previously into climate change assessments studies^34,35^, permafrost sensitivity studies^40^ and superimposed ice simulations^41^ as well as the simulation of snow storage^42^.Full description of the SNOWPACK model is available in^32,43,44^, and^45^.

Alpine3D includes various modules to compute energy fluxes to simulate snow cover development. It encloses, vegetation cover, snow transport and radiation transfer as well as runoff module. The basic strength of Alpine3d is to provide realistic snow cover development in the mountainous topography with steep slopes^39^. It has been applied for assessment of snow and water resource in mountain catchments^24^ and predictions of future snow under climate change scenarios^35^ as well as glacier surface modeling^41^. Alpine3D accepts a variety of input options including inputs from other meteorological models or remote sensing equipment’s. MeteoIO library includes various interpolation schemes^46^ is part of Alpine3D model and allows to distribute the input data from meteorological weather stations to the catchment DEM grid through spatial interpolation and extrapolation while maintaining consistency of data. Alpine3D mainly require landscape, snow cover and soil as well as meteorological data to model snow accumulation and ablation dynamics in the mountainous watershed. Snow development can be visualized via SN_GUI tool^47^ at allocated points by creating special point file via built in view.sh tool. Hydrological discharge can be computed using PREVAH hydrological scheme^48^ and is available together with Alpine3D.The precipitation and melt water for each cell of the domain grid or each sub catchment defined by the user are collected in grid files and can be processed with the PREVAH hydrological model or with any other external hydrological routine. Full description of the Alpine3D is available in^39^ and ^49^.

## Material and Methods

This section discusses availability and preparation of model input and validation data at the model optimization site and main study site. Both static and dynamic data sets were used. Static data sets include spatial data such as Digital Elevation Model grid, Land Cover, catchment area, soil conditions etc. Whereas, dynamic data sets comprises of meteorological and hydro-meteorological data as well as snow depth data.

### Model optimization data

Model was optimized at Urdukus weather station which is located at a distance of 164 km SE of Passu glacier having an altitude of 3926 m a.s.l as shown in Fig. 1. It is the nearest available station in the vicinity of Passu glacier with minimum elevation difference between them. Moreover, Urdukus weather station was the only closest station available in the Gilgit-Baltistan region where required meteorological and snow depth data sets were available that are necessary to run the SNOWPACK model. The glaciers flowing from K2, Broad Peak, and Gasherbrum I and II plus many others are joining at Concordia and Urdukus and flowing out to the Baltoro glacier attaining 60 km length^50^.

In this region, EV-K2-CNR Association (Italian Research Council) has three automatic weather stations (AWTs) operative under SHARE project (Stations at High Altitude for Research on the Environment). These stations are providing unique information that is useful to characterized meteorological condition of Baltoro Glacier. Urdukas weather station records all the standard meteorological variables except solar radiation, at an hourly frequency since, 2004. In 2011, radiation sensors were also installed at this station to observe the variations in the short-wave and long-wave radiation budget. Table 1 show the available data sets at AWT- Urdukus used in SNOWPACK simulation.

**Table 1:** Description of the available meteorological variables and period at Urdukus station.

Meteorological Variable	Unit	Observation Frequency	Height of Instrument (m)	Period (years)
Air Temperature	°C	1 hour	2	2006–2012
Relative Humidity	%	1 hour	2	2006–2012
Precipitation	mm/hr	1 hour	1.5	2006–2012
Wind Speed	m/s	1 hour	5	2006–2012
Wind Direction	0° to 360°	1 hour	5	2006–2012
Solar Radiations	W/m^2^	1 hour	2	2011–2012
Snow depth	m	1 hour	2	2012

It is important to note that snow depth data was only available for the year 2012 to validate the model performance. Hence, the optimization of model became restricted to only year. Moreover, precipitation data for the year 2012 held small gaps with no data. Thus, data was linearly interpolated keeping in view the average precipitation value for those time steps in the year 2011. Consequently, ideal performance of the SNOWPACK model at Urdukus could not be expected.

### Spatial gridded data

**Figure 2:**
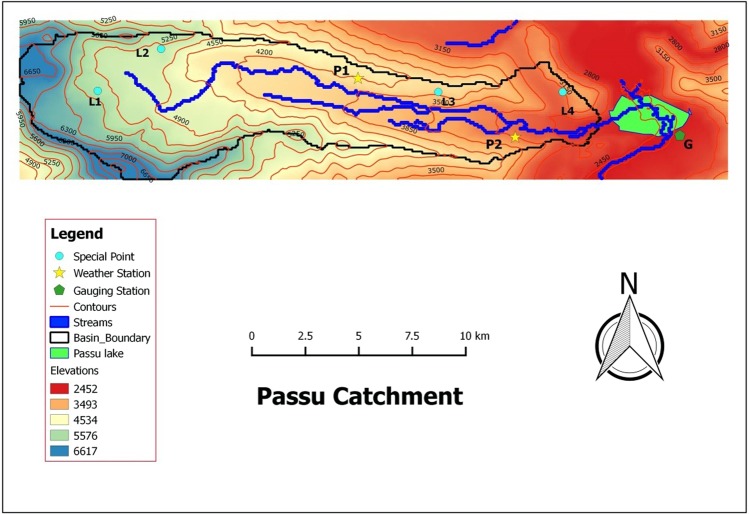
Original clipped DEM and delineated Passu Catchment used in Alpine3D simulations (*This map was made using QGIS software (version 2.18)*.

Spatial data comprises of digital elevation model (DEM) and land cover detail of the main study site. The resolution of the DEM grid decides the spatial resolution of the model simulations. Every cell whose DEM is no data is not considered by all the modules of Alpine3D. DEM grid at the resolution of 90 m was downloaded for the study region from http://srtm.csi.cgiar.org/. It was then clipped to exclude the area outside our interest using Geographical Information System (GIS) software shown in Fig. 2.

Boundary of Passu catchment was defined by filling the unnecessary cells with no-data in order to reduce unnecessary model computational time. Landsat TM 30 × 30 m resolution data set for the years 2009 and 2010 was used to extract land-cover of the study area and monisacked with DEM to identify the land cover distribution of the study catchment at initial stage. Nevertheless, resolution of the data was unable to identify clearly the land cover for such a small-scale catchment. Consequently, land cover detail is manually provided considering previous literature and assistance from google map. Passu is a clean glacier with no considerable debris^25^. Land-cover in the sub-basin is mainly comprised of snow and glacier (73%) and barren rock (27%) with no vegetation cover.

Based on the land cover classifications each cell was then assigned with soil condition and initial snow profile (no snow conditions are assigned to start the simulations). Keeping in view simulation period of one year, glacier depth is assumed as 30 m. All the data is then converted into alpine3D executable numerical format in GIS.

### Meteorological data

Alpine3D requires at least six standard meteorological variables to successfully simulate the catchment weather conditions and snow development. Input data must be comprised of temperature (TA), relative humidity (RH), wind speed (VW), precipitation (P) or snow heights (HS), incoming short wave radiation (ISWR) or incoming long wave radiation (ILWR). The model can only be driven on an hourly resolution and so the data should be either hourly or resampled to an hourly resolution.

Water and Power development Authority (WAPDA) has three high altitude weather stations operative in the Hunza watershed at Khunjrab (Khu), Ziarat (Zia) and Naltar (Nal) shown in Fig. 1. Description of the meteorological stations and variables used in this experiment along with their locations and altitude are given in Table 2. For the accuracy in Alpine3D simulations, it is recommended that the selected stations are well distributed over the study domain with minimum elevation difference in order to properly compute elevation gradients. The above stations are therefore selected due to their specific locations and data from above three stations have been converted into Alpine3D suitable format (ASII) for the year 2014–16. These stations record all the standard meteorological variables that are necessary to run the Alpine3D model. Moreover, these stations are equipped with precipitation gauges that measure the total precipitation (solid + liquid) at hourly rate. It is important to mention that inconsistency of data has been observed for certain variables at specific times during consistency check. Which was corrected by applying average trend using standard consistency method; however, uncertainty in this data cannot be completely ignored.

**Table 2:** Detail of Meteorological and Hydro-meteorological data availability and resource authority.

Weather Stations	Station ID	Altitude [m]	Coordinates	Meteorological Variables	Source
Khunjrab	KHU	4730	36°51′0.00″N 75°24′0.00″E	TA_max_, TA_min_, RH ISWR, ILWR,WS, WD, P_sum_	WAPDA
Ziarat	ZIA	3669	36°49′59.88″N 74°25′59.88″E	TA_max_, TA_min_, RH ISWR, ILWR,WS, WD, P_sum_	WAPDA
Naltar	NAL	2858	36°13′0.12″N 74°16′0.12″E	TA_max_, TA_min_, RH ISWR, ILWR,WS, WD, P_sum_	WAPDA
Passu_Upper	P1	4227	36°28′40.99″N 74°47′43.86″E	TA,RH,ISWR,WS, WD, P_liq_	PMD
Passu_Lower	P2	3162	36°27′15.40″N 74°50′39.13″E	TA,RH,ISWR,WS, WD, P_liq_	PMD
Urdukus	Urd	3926	35°43′41.00″N 76°17′10.00″E	TAmax, TAmin, RH ISWR, ILWR,WS, WD PSum	EV-K2-CNR
Passu Guaging Station	G	2641	36°27′27.86″N 74°52′56.56″E	Water surface level (D) and Flow discharge (Q)	WAPDA-ICIMOD

Whereas, PMD = Pakistan meteorological department, WAPDA = water and power development authority. ICIMOD = International Centre for Integrated Mountain Development.

In 2011, Pakistan Meteorological Department, Islamabad (PMD) in cooperation with International Centre for Integrated Mountain Development (ICIMOD) has installed two automatic weather stations (AWTs) P1 and P2 shown in Fig. 2. These AWTs monitor gradient flows and the ablation rates at the Passu glacier. These stations record hourly measurements standard meteorological variables except solid precipitation. Although, AWSs are equipped with precipitation gauges that are capable to measure the total precipitations but was not recorded for the particular year due to technical dysfunction of the gauge. The hourly data has been received from PMD to validate the interpolations of meteorological parameters at Passu catchment for the year 2015.

### Hydrological data

Hydro-meteorological monitoring station is operational since 2010, at the Passu lake. This measurement equipment was installed by WAPDA in October 2010 at latitude 36°27′28″N and longitude 74°52′57″E. The equipment comprises of Pressure Transducer and Data Logger. Gauging site is located about 1 km from the bridge Karakoram Highway just downstream of the channel issuing from the lake formed by the end moraines of Passu glacier.

**Figure 3:**
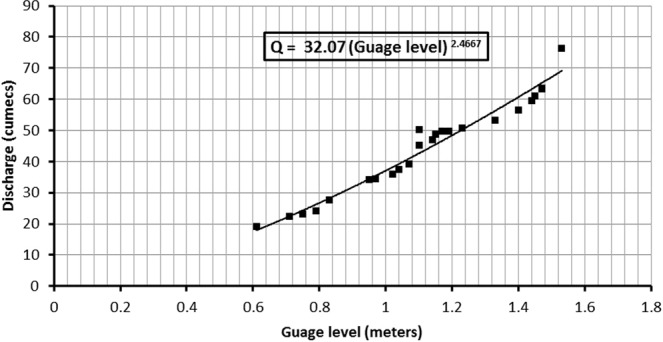
Rating curve generated at Passu gauging station for the year 2015.

Twenty three discharge measurements along with recorded water levels for the year 2015 were utilized in this experiment. The available discharge measurements were plotted against the corresponding stream levels and power curve was drawn shown in the Fig. 3. Stream levels range between 0.10 m to 1.8 m by increments of 0.04 meters. Eq. 3 was obtained to express the relationship between discharges and stream levels.

3 $${\rm{Discharge}}=32.07\ast {(\mathrm{Stream}\mathrm{level})}^{2.4667}$$

**Figure 4:**
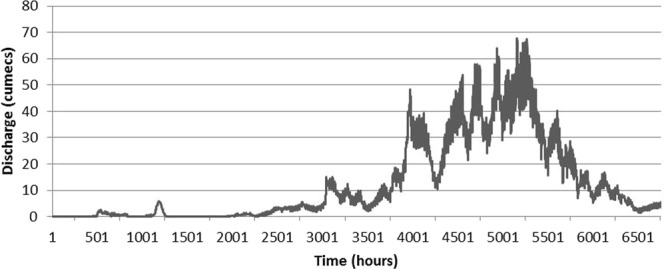
Hourly hydrological discharge at Passu Catchment for the year 2015.

Whereas, discharge is in cumecs and stream level is in meter. The above relationship was used to develop the rating table and hydrological discharge is plotted for the year 2015 shown in Fig. 4.

Hydrological discharge data shown in Fig. 4 above was compared with the modeled discharge and Nash Sutcliff efficiency was calculated to check model performance by using Eq. 4.

4 $${\rm{N}}{\rm{a}}{\rm{s}}{\rm{h}}\,{\rm{S}}{\rm{u}}{\rm{t}}{\rm{c}}{\rm{l}}{\rm{i}}{\rm{f}}{\rm{f}}\,{\rm{e}}{\rm{f}}{\rm{f}}{\rm{i}}{\rm{c}}{\rm{i}}{\rm{e}}{\rm{n}}{\rm{c}}{\rm{y}}=1-\frac{{\sum }_{{\rm{i}}=1}^{{\rm{n}}}\,{({{\rm{Q}}}_{{\rm{i}}({\rm{o}}{\rm{b}}{\rm{s}}{\rm{e}}{\rm{r}}{\rm{v}}{\rm{e}})}-{{\rm{Q}}}_{{\rm{i}}({\rm{m}}{\rm{o}}{\rm{d}}{\rm{e}}{\rm{l}})})}^{2}}{{\sum }_{{\rm{i}}=1}^{{\rm{n}}}\,{({{\rm{Q}}}_{{\rm{i}}({\rm{o}}{\rm{b}}{\rm{s}}{\rm{e}}{\rm{r}}{\rm{v}}{\rm{e}})}-\bar{{\rm{Q}}})}^{2}}$$

Whereas, Q represent discharge and $\bar{{\rm{Q}}}$ represents mean observed discharge.

### Model optimization and configuration setup

Model optimization was carried out at Urdukus station, Hunza watershed, Pakistan due to the following reasons;

- This particular weather station measures the required six meteorological variables that are necessary to run the model.
- This is the only station where solid precipitation and snow depth data were available to use for input and validation of the model.
- Urdukus station and the main study catchment, Passu are not too far from each other and thus retains comparable local weather conditions.

SNOWPACK model, which is included as a sub-module in Alpine3D model was used for this optimization. SNOWPACK is a process based model and does not require calibration but it contains few parameters that can be adjusted with respect to the observations at the given site for high performance. The most important parameter adjustments include coordinate system, boundary conditions, mode of model, stability corrections, canopy detail, and precipitation correction factor. For this study, above parameters were optimized through configuration setup of the model (config.ini file). Final setup that could be achieved is shown in Table 3. The objective function of this optimization was to achieve minimum error between the observed and simulated snow cover development through the use of performance evaluators namely Nash-suttcliff effeciency (NSE), Mean squared error (MSE), Root mean square error (RMSE). These performance indices are described as following in the Eqs 5–7.

5 $$RMSE=\sqrt{\frac{{\sum }_{i=1}^{n}\,{({S}_{i}(mod)-{S}_{i}(obs))}^{2}\,}{n}\,}$$

6 $$MSE=\frac{1}{n}\sum _{i=1}^{n}\,{({S}_{i}(obs)-{\bar{S}}_{i}(mod))}^{2}$$

7 $$NSE=1-\frac{{\sum }_{i=1}^{n}\,{({S}_{i}(obs)-{S}_{i}(mod))}^{2}}{{\sum }_{i=1}^{n}\,{({S}_{i}(obs)-\bar{S})}^{2}}$$

Where, S(obs) is the observed, S(mod) is the simulated and $\bar{{\rm{S}}}$ is the mean observed snow depth. In the above equations “n” represents the simulation period in days. Crucial model parameters and adjustments made are provided in Table 3. These adjustments were checked by comparing the observed and simulated snow depth at Urdukus weather station.

**Table 3:** SNOWPACK model parameter adjustments at Urdukus weather station.

Parameter	Symbol	Adjustment	Detail
Coordinate System	COORDSYS	UTM	Coordinate system used to specify locations in the model
Model Boundary Condition	CHANGE_BC	Neumann	Two boundary conditions are available at snow-atmosphere interface. Neumann or Dirichlet
Roughness length	ROUGHNESS_LENGTH	0.002	Mass of an element that is neither changed by phase changes nor densification
Mode of model	SW_MODE	Incoming	Based on available data. Incoming short wave radiation or both incoming short and longwave radiations.
Enforced measure snow height	ENV_Meas_Snow_Depth	False	Uses measured precipitation or snow height as input
Canopy detail	CANOPY	False	Canopy detail at study area or no vegetation
Input Precipitation data treatment	PSUM	Accumulate	PSUM is accumulated at hourly temporal resolution or raw.
Atmospheric Stability	ATMOSPHERIC_STABILITY	Monin-Obukhov^44^	Various stability-correction schemes are available to consider temperature inversions on snow and glacier surface.
Geothermal heat flux	GEO_HEAT	0.06	Depends on type of soil
Soil albedo Snow albedo Ice albedo	Soil_Albedo Snow_Albedo Ice_Albedo	0.2–0.3	Depends on type of soil Computed at each time step by the model Fixed^58^

## Results and Discussion

In this section model optimization process through SNOWPACK model and simulation results at the main study site via Alpine3D have been presented and discussed. Performance of the model interpolation schemes for the distribution meteorological data have been checked through statistical performance evaluator and presented in tabulated form. Simulated snow depth at various locations of the glacier surface has been shown. Graphical flow composition and hydrological discharge comparison have been shown and the reasons for overestimations have been discussed.

### Performance check at urdukus

**Figure 5:**
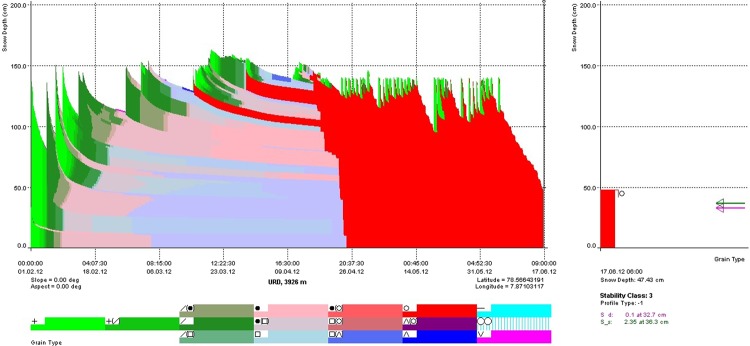
Simulated snow development for the year 2012, at Urdukus weather station, shown in SN_GUI interface.

The parameters described in Table 3 are adjusted by several trials simulations with respect to the agreement between observed and simulated snow depths. Graphical user interface (sn_gui) was used for visualization of simulated snow depth^47^. Figure 5 shows the original snowpack development by the SNOWPACK model (showing grain classifications in various colors) at Urdukus station for the year 2012. Different colors represent grain types based on age and metamorphism process of snow. Green represent fresh snow, blue represent the faceted and red represent the melted snow grains^43^. Snow melting is started in the month of March and complete snowpack is melted out in the month of June 2012. During the month of the June, 2012 fresh snow and melting occur simultaneously whereas melting is high in July and represents high positive energy flux.

Performance of the model was evaluated by computing RMSE, NSE and MSE using Eqs 4–6. Final RMSE = 1.24 and NSE = 0.87 values were not highly satisfactory however; MSE was low i.e. 0.09 which shows an acceptable relationship between measured and simulated snow depths at Urdukus. Keeping in view the data quality discussed in above. it was decided to continue the experiment with the same setup of the configuration file in Alpine3D for Passu catchment.

**Figure 6:**
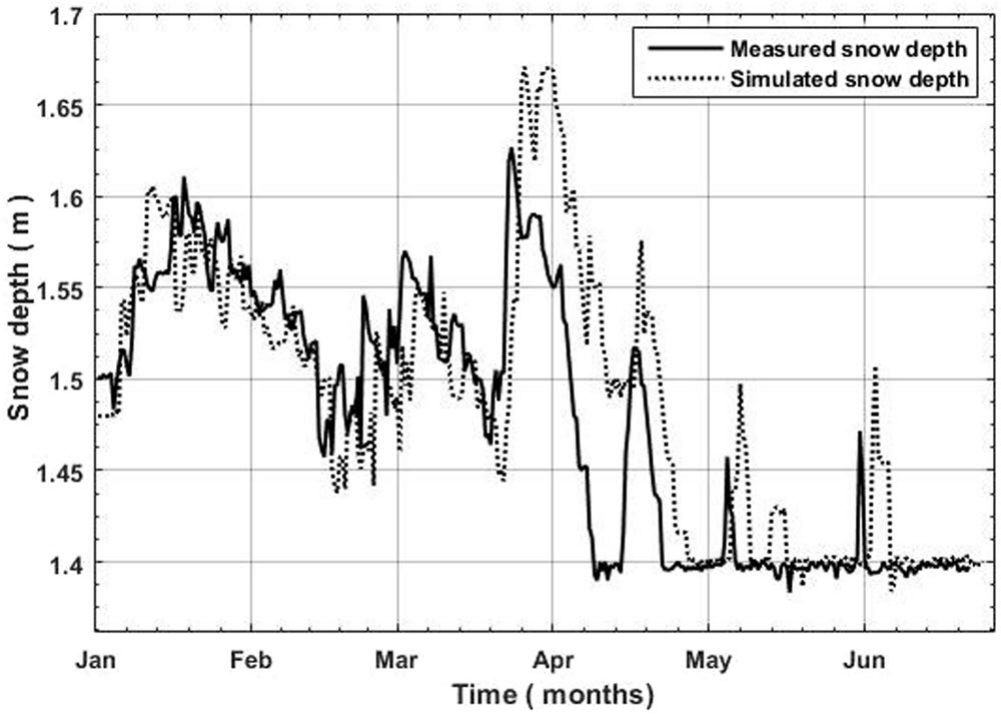
Measured and simulated snow development season at Urdukus weather station for the year 2012.

The graphical comparison of measured and simulated snow depths at a local weather station Urdukus is presented in Fig. 6. As expected, the results were not highly accurate and suggest that model under predicted snow accumulation period, from Jan-March and over-estimated the melting during snow melting season in May and June. The overestimation of solid precipitation may be associated with the average precipitations estimate used as an input which might not be the full representative of real snow development conditions for the year 2012. We are not able to validate the other choices that were made for the final setup of the model due to the quality of input data utilized. We proceeded with such performance because at Passu glacier we didn’t even had the snow depth data to evaluate the final simulated snow cover by the model. The model performance could only be evaluated by comparing the hydrological discharge after the distributed application of the SNOWPACK model by applying Alpine3D. We acknowledge that model optimization at Urdukus station might not be the full representative of the Passu catchment weather conditions. But this station was our only choice as explained in above

### Distribution of meteorological variables to the catchment grid

The meteorological variables including TA, ISWR, RH, WS, WD and Psum were distributed to the catchment grid using interpolation schemes of MeteoIO library of Alpine3D. The values of distributed variable for the year 2015 were then compared with the observed data at local weather station (P1) of Passu catchment shown in Fig. 2. The model performance was evaluated by checking the statistical parameters such as mean error (ME), root mean square error (RMSE), and Nash-suttcliff effeciency (NSE) and final values are presented in Table 4.

**Table 4:** Statistical performance of modeled distributed meteorological variables and snow depths with observed data at validation station Passu.

Catchment (Period)	Meteorological variables	Units	Time step	NSE	RMSE	ME
Passu catchment (Year 2015)	Air temperature	^0^C	hourly	0.96	1.39	0.22
	Relative humidity	%	hourly	0.79	10.09	0.43
	Inc. short wave radiation	w/m^2^	hourly	0.91	76.11	15.3
	Inc. long wave radiation	w/m^2^	—	—	—	—
	Acc. liq. Precipitation	Mm	daily	0.50	67.3	13.2
	Wind speed	m/s	hourly	0.19	2.7	1.42

**Figure 7:**
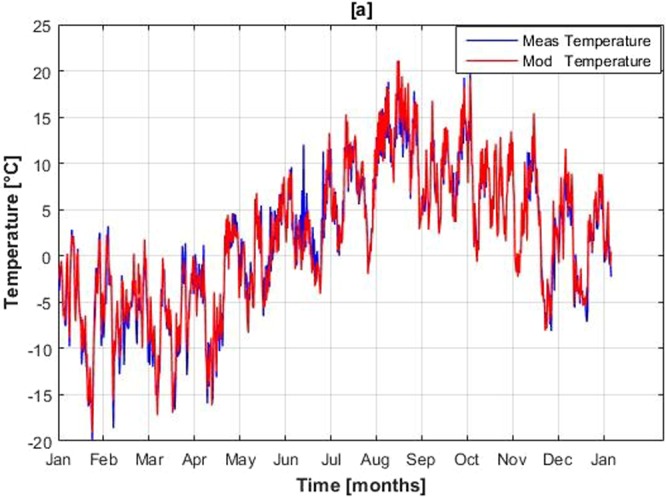
Measured and modeled temperature at local weather station, Passu Catchment for the year 2015.

**Figure 8:**
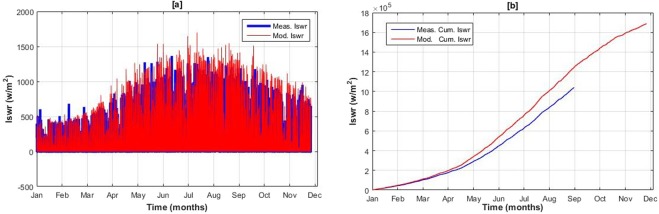
Hourly Observed and modeled Incoming shortwave radiation at local weather station, Passu Catchment for the year 2015.

Temperature, relative humidity and incoming shortwave radiation are distributed well by the model and suggest a good correlation with the observed data at P1. Graphical comparison of observed and simulated temperatures and incoming shortwave radiation are shown in the Figs 7 and 8 respectively. Slight overestimation in ISWR can be seen in the cumulative graph. This overestimation may raise the net heat flux into the snowpack and may increase the melt slightly.

**Figure 9:**
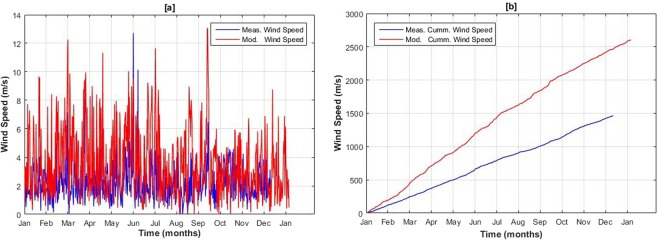
12^th^ Hourly Observed and modeled wind speeds (**a**) and cumulative wind speed (**b**) at Passu validation station for the year 2015.

On the other hand, wind speeds were considerably overestimated at validation station showing NSE = 0.19. Figure 9(a) shows the modeled and observed wind speed at the LWS, Passu. Overestimation of wind speed can also be observed in the cumulative plot in Fig. 9(b).

Wind speed is a highly variable parameter especially at high altitudes complex terrains and depends on the local conditions of the specific catchment^51^. Computation of realistic wind speed is therefore very difficult due to its temporal and spatially varying nature in the mountainous regions^52–54^. To correct the modeled wind speeds at the Passu catchment, data has been reexamined. It was found that (Khu) weather station contains high wind speed values and may not be the representative of the Passu catchment. Thus, model was driven with restricted wind speed inputs from this particular station. Nevertheless, no considerable improvement was noted. This overestimation of wind speed may cause unequal snow deposition and distribution in different parts of the catchment due to wind snow transport as investigated by^55,56^.

**Figure 10:**
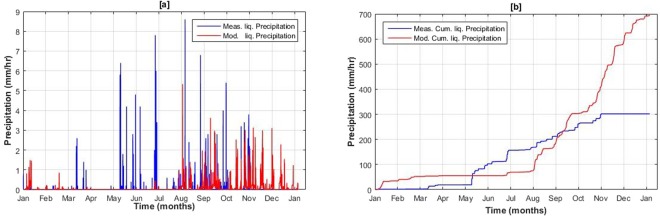
12^th^ Hourly observed and modeled liquid precipitation at local weather station, Passu catchment for the year 2015.

On the other hand, due to dysfunctional precipitation gauge, solid precipitation data was not available at both P1 and P2 stations at Passu catchment. However, plot of observed and modeled liquid precipitation is presented in Fig. 10(a,b) as model can discriminates between liquid and solid precipitation based on temperature. Cumulative plot depicts considerably high disagreement between observed and measured data which confirms poor correlation values NSE (0.5) and MSE (13.2). It suggest that the inverse distance weighing interpolation algorithm with a constant lapse rate used for liquid precipitation distribution might not be the best option in a steep and complex terrains at high altitudes.

Alpine3D itself computes ILWR based on ISWR, RH and TA by considering Konzelmann parameterization^57^. Incoming long wave radiations were not available at this station for validation of the outputs.

### Modeled snow depths over the glacier surface

**Figure 11:**
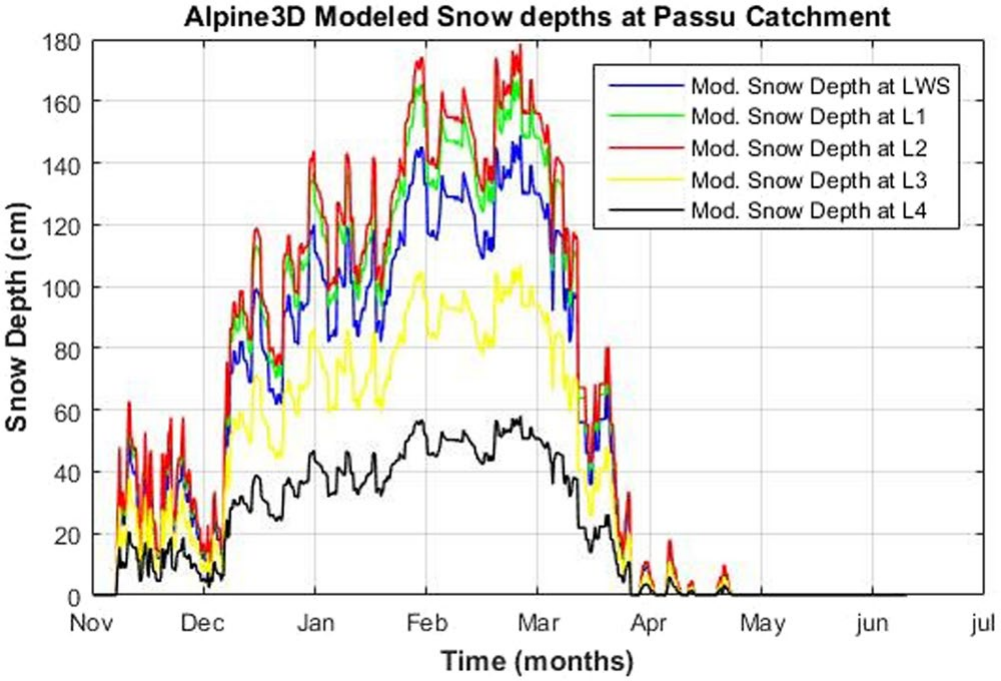
Comparison of modeled snow depths at LWS (P1) and special locations L1, L2, L3 and L4.

Local weather stations located nearby Passu glacier lacking snow depth measurement sensors. We are therefore not able to comment on the performance of the model for snow cover development in this particular case. However, simulated snow depths by Alpine3D can be visualized at P1 and initially specified points. These points are allocated, at various points of interest over the glacier surface, during creation of point file at the time of data preparation discussed above. In this particular case we have selected two special points above and two below the approximate equilibrium line (firn line) which is based on concavity and convexity of the contour lines. L1 (5414 m) and L2(5172 m) are in the upper part of the glacier (accumulation zone) and hence show more snow depths than L3(3930 m) and L4(3064 m) which are at the lower elevation (ablation zone) of Passu glacier shown in Fig. 2. Graphical comparison of simulated snow depths at these special locations are shown in Fig. 11.

Non- availability of snow depth data at this site to validate the model performance limits the performance check of the model. We acknowledge that any over or underestimation in snow depths can incur significant in-accuracy in the Alpine3D hydrological discharge estimates. The experiment carried on to validate the simulated flow at the outlet of Passu lake with an anticipation that the use of energy balance model and its optimization will foster the acceptable correlation.

### Hydrological discharge and flow composition

**Figure 12:**
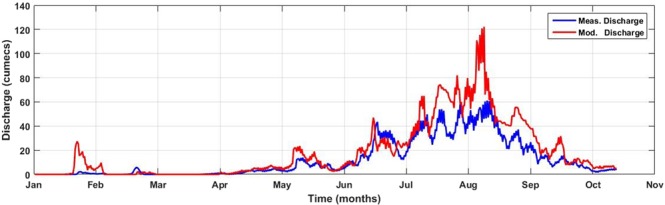
Twelfth hourly averaged observed and modeled hydrological discharge at Passu gauging station for the year 2015.

Modeled hydrological discharge computed by PREVAH hydrological scheme discussed above was compared with the derived discharge obtained from the rating curve. Figure 12 illustrates the graphical comparison between twelfth hourly averaged, measured and modeled flow rates at Passu gauging station (G) for the year 2015.

Alpine3D reproduced the snow melt dominated runoff period (April to July) satisfactorily with slight overestimation and underestimation. However, sub-optimal performance of the model has been observed during glacier melt dominated runoff period (Aug to October) in the year 2015 (Fig. 12). There are at least two possible reasons why the model shows this behavior. First, Snow and ice are considered in the same way by the energy-balance melt model, the only difference being their respective albedo (Table 3). Snow albedo is computed at each time step with the albedo sub-module of SNOWPACK^44^, whereas, Ice has a fixed albedo of 0.3^58^. This simplified approach might have forged the model to compute excessive melts during glacier melting season. In future, Alpine3D code may be be modified to compute robust dynamic ice albedo as in^59^. Second reason is the abnormal high wind speed computations discussed above, which enhances the turbulent fluxes over the snow surface. Turbulent fluxes impact directly the energy-balance and raise the heat flux enters into the snowpack leading to the melt overestimation. Moreover, uncertainty of input data might have played a part leading to this overestimation.

However, overall performance of the model met satisfactory threshold with the Nash Sutcliff efficiency of 0.54 (Table 5). As per NSE criteria, simulation results are considered very good for values of NSE above 0.75, good for NSE values between 0.65 to 0.75 and satisfactory for NSE values between 0.50 and 0.65^60^. Refrained excellent performance of the model is more likely to be associated with the input data quality discussed above, or non-accurate precipitation distribution eventually leading to bias snow cover development. Moreover, uncertainty due to the use of flow validation data which was derived from the rating curve cannot be ignored.

**Table 5:** Comparison of total observed and modeled hydrological discharge along with flow composition.

Measured Discharge [m^3^]	Modeled Discharge [m^3^]	Percentage Error	Total Difference	NSE	Snow [%]	Ice [%]	Rain [%]
**Discharge Passu Lake (Jan-Dec2015)**							
1.62 × 10^5^	2.13 × 10^5^	23%	71226	0.54	53	38	9

**Figure 13:**
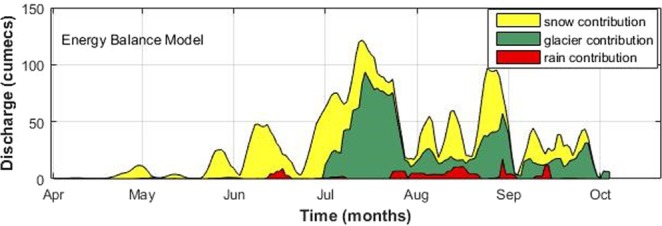
Three-days averaged simulated flow composition showing liquid precipitation, snow melt and glacier melt contribution at Passu outlet in the year 2015.

Total runoff is contributed by rainfall on land, snow, and glacier melt. Snowmelt runoff accounted for a large proportion of Passu glacier runoff (53%), whereas glacier melt runoff accounted as 38% (Table 5). Rainfall-induced runoff had the least contribution of only (9%). Figure 13 confirms the share of excessive glacier melt contribution in the total runoff during the period of glacier melting season.

However, total hydrological discharge computed by Alpine3D was in good agreement and only 1.3 times to the total measured discharge with a percentage error of 23%. The model performance is considered “Satisfactory” if the percentage error falls between 15–25%^60^. These results encourage the future applicability of this model in Pakistan and suggest that better efficiency can be achieved if it is driven with quality input and validation data.

## Conclusion and Recommendations

SNOWPACK and ALPINE3D energy balance models have been applied for the first time in Pakistan to assess the hydrology of Passu catchment located in Hunza Watershed, Pakistan. The aim was to test the performance and feasibility of these models in order to quantify the hydrological discharge in high altitude mountainous catchments of Pakistan. The results demonstrate temporal scatter between measured and modeled flow rates with NSE of 0.54 and just meet the satisfactory performance threshold. Flow composition analysis reveals the simulated lake flow is comprised of snowmelt, glacier melt, and rainfall runoff. Snowmelt runoff accounted for a large proportion of Passu catchment runoff (53%); whereas glacier melts runoff contributed 38% and rainfall-induced runoff had the smallest contribution (9%). Sub-optimal performance of model in glacier melting season has been observed mainly due to the overestimation of wind speed and biased precipitation distribution inputs. Moreover model does not compute robust albedo for ice surface which might lead to the overestimation during glacier melting season. Non-availability of the enough and accurate total precipitation and snow depth data is the weak part of this study. Although, careful selection of the model parameters and interpolation schemes has a considerable affect over NSE values. But, we adjudge that uncertainty of the input data and poor validation data were the biggest factors of the low performance. However, model was able to reproduce the total lake flow satisfactorily, showing only 1.3 times increase as compared to the measured discharge for the year 2015.

It is comprehended that this model system can be successfully applied at high altitude glaciated catchments of Pakistan to study hydrological processes. It also imply that performance of this model system can be enhanced provided if, quality input data free from bias is available for optimization and simulations. Prior optimization and adjustment of the model at a site with similar local conditions enhance the model accuracy. Careful selection of weather stations which are representative of the catchment local weather plays a vital role in the accuracy. The capabilities of this model system and reasons of its limited performance have been discussed in this paper. Its application in the Pakistani Alps is highly recommended for understanding glacio-hydrological processes and testing it with long term data and robust ice albedo. This study also encourages the use of SNOWPACK model for future mass and energy balance studies and to explore its potential applications in the context of climate change as well as avalanche warning system in Pakistan. The importance of strengthening the existing network of hydro-meteorological monitoring and increasing the lengths of record should not be ignored. It also emphasizes on the installation of more weather stations equipped with snow depth sensors and precipitation gauges that are capable to measure solid precipitation in the accumulation zone of high altitude glaciers. The data sets and model optimization technique used in this study for parameter selection can increase the internal consistency of the hydrological models. In present times, most of the hydrological modeling studies utilize remotely sensed data sets. However, short-term specific monitoring programs in the mountainous Alps to quantify water budget and actual status of the cryosphere can be a meaningful substitute of long-term observations.

## Data Availability

Data will be made available upon request.
